# Characterization of Colombian departments based on climatic factors, infrastructure, basic service access, and dengue incidence rate

**DOI:** 10.7705/biomedica.7865

**Published:** 2025-11-27

**Authors:** Jennyfer Portilla, José Tovar-Cuevas, Diego Manotas

**Affiliations:** 1 Escuela de Ingeniería Industrial, Facultad de Ingeniería, Universidad del Valle, Cali, Colombia Universidad del Valle Universidad del Valle Cali Colombia; 2 Escuela de Estadística, Facultad de Ingeniería, Universidad del Valle, Cali, Colombia Universidad del Valle Universidad del Valle Cali Colombia

**Keywords:** Aedes aegypti, dengue, socioeconomic factors, multivariate analysis, public health, Aedes aegypti, dengue, factores socioeconómicos, análisis multivariante, salud pública

## Abstract

**Introduction.:**

Dengue is an endemic disease in Colombia, with spatial variations influenced by climatic, socioeconomic, and basic service access factors. Territorial characterization based on these determinants supports a better understanding of disease distribution and enables the design of more effective control strategies.

**Objectives.:**

To identify groups of departments in Colombia based on the relationship between dengue incidence rates and climatic, socioeconomic, and basic service access factors in 2023.

**Materials and methods.:**

Data were collected from the *Instituto Nacional de Salud* of Colombia, the *Encuesta Nacional de Calidad de Vida*, and satellite sources, such as ERA5 and CHIRPS. Variables related to access to basic services (drinking water, sewage, and waste collection), housing deficit, temperature, precipitation, and the normalized difference vegetation index (NDVI) were analyzed. A multiple factor analysis was applied to reduce dimensionality, followed by hierarchical clustering and self-organizing maps to identify department groupings.

**Results.:**

Three groups of departments with distinct characteristics were identified. The most vulnerable group (group 3) showed an average incidence rate of 1,046.87 cases per 100,000 inhabitants, associated with extreme housing deficits, limited access to basic services, and climatic conditions favorable for vector proliferation.

**Conclusions.:**

The analysis identified key territorial patterns in dengue incidence and highlighted the influence of structural factors on disease transmission. These findings provide a foundation to strengthen public policies and design more targeted prevention and control strategies in the most vulnerable regions.

## Resumen

**Introducción.:**

El dengue es una enfermedad endémica en Colombia cuya incidencia varía espacialmente debido a factores climáticos, socioeconómicos y de acceso a servicios básicos. La caracterización territorial basada en estos factores determinantes permite comprender la distribución de la enfermedad y orientar estrategias de control más precisas.

**Objetivo.:**

Identificar grupos de departamentos de Colombia según la relación entre las tasas de incidencia de dengue y los factores climáticos, socioeconómicos y de acceso a servicios básicos en el 2023.

**Materiales y métodos.:**

Se recopilaron datos del Instituto Nacional de Salud, la Encuesta Nacional de Calidad de Vida y fuentes satelitales, como ERA5 y CHIRPS. Se analizaron las variables relacionadas con el acceso a los servicios básicos (agua potable, alcantarillado y recolección de basuras), el déficit habitacional, la temperatura, la precipitación y el *Normalized Difference Vegetation Index* (NDVI). Se hizo un análisis factorial múltiple para reducir la dimensionalidad y, posteriormente, se identificaron conglomerados (*clusters*) jerárquicos y mapas autoorganizados.

**Resultados.:**

Se identificaron tres grupos de departamentos con características diferenciadas. El grupo más vulnerable (grupo 3) registró una tasa de incidencia promedio de 1.046,87 casos por cada 100.000 habitantes, asociada con un déficit habitacional extremo, acceso limitado a los servicios básicos y condiciones climáticas favorables para la proliferación del vector.

**Conclusiones.:**

El análisis permitió identificar patrones territoriales clave en la incidencia del dengue y evidenció la influencia de otros factores estructurales en la propagación de la enfermedad. Estos hallazgos pueden servir como base para fortalecer el diseño de políticas públicas y orientar estrategias de prevención y control más focalizadas en las regiones más vulnerables.

According to the World Bank Group, Colombia is a country with high geographical and climatic diversity, shaped by the Andes Mountains, tropical rainforests, and extensive lowland plains. Its territory covers more than 1.1 million square kilometers, with coastlines on the Pacific Ocean and the Caribbean Sea ^1^. The Andes cross the country from south to north, forming three major mountain ranges -Western, Central, and Eastern- that reach elevations above 5,000 m and generate distinct climatic zones, ranging from warm lowlands to snow-capped peaks. This topographical diversity supports a wide range of ecosystems and contributes to significant variability in climate, precipitation, and land use across regions.

Despite being classified as an upper-middle-income country, Colombia exhibits marked socioeconomic inequalities. Wealth is highly concentrated in big cities, such as Bogotá, Medellín, and Cali, whereas many rural and peripheral departments, including those along the Pacific coast (*e.g*., Chocó), face chronic underdevelopment, limited infrastructure, and persistent poverty.

From a climatic point of view, Colombia is recognized as a megadiverse country, with ecosystems ranging from moors and glaciers in the Andes to mangroves, tropical rainforests, wetlands, and coral reefs. Its complex topography gives rise to three main thermal zones: the hot zone (below 1,000 m), the temperate zone (between 1,000 and 2,000 m), and the cold zone (above 2,000 m). Rainfall patterns are similarly diverse: while the Andean region experiences two rainy seasons (April to June and October to December), the Caribbean region has only one (May to October). In addition, Colombia is highly vulnerable to interannual climate variability influenced by El Niño-Southern Oscillation (ENSO), which alternates between periods of drought and intense rainfall ^1^.

Dengue is a viral disease caused by four serotypes and transmitted mainly by infected female mosquitoes of the Aedes aegypti species and, to a lesser extent, by *Aedes albopictus*^2^. It is endemic in more than 100 countries, primarily in tropical and subtropical regions such as South America, Central America, and the South Pacific. Currently, more than 40% of the world’s population lives in areas at risk ^3^. According to the Health Information Platform for the Americas of the Pan American Health Organization (PAHO) ^4^, 4,594,823 cases were reported in the Americas in 2023, underscoring the urgent need to implement effective prevention and control strategies. In Colombia, incidence varies historically between departments, as documented in the *Boletín Epidemiológico Semanal* of the *Instituto Nacional de Salud*, an essential source to monitor and analize the evolution of the disease at the national level.

Studying the geographic distribution of dengue in Colombia is crucial to identify patterns in incidence rates and the most vulnerable departments. A perspective that considers climatic factors, infrastructure, and access to basic services -such as drinking water, sewage, and waste collection- allows for a more comprehensive understanding of the factors that influence transmission.

An example of this approach is a study in Costa Rica ^5^, which highlights the relationship between dengue and social and environmental determinants. That study notes that socioeconomically vulnerable populations face a higher risk, while inadequate water storage and deficits in water supply and waste collection services facilitate vector proliferation. Similarly, in Guatemala, a study indicates that poverty and lack of access to basic services, including safe water and sanitation, contribute significantly to the spread of dengue ^6^. These findings reinforce the importance of considering social and environmental determinants in the design of policies for the prevention and control of the disease.

To address territorial heterogeneity in dengue incidence, this study applies multiple factor analysis to reduce the dimensionality of climatic, socioeconomic, and infrastructure-related variables, complemented by hierarchical clusters and self-organizing maps to identify patterns of similarity between departments. These methodologies enable a more accurate classification of Colombian territories according to structural determinants associated with dengue transmission. The analysis identified three distinct groups, among which the most vulnerable exhibited the highest average incidence rate, accompanied by high housing deficits, limited access to basic services, and hot and humid climatic conditions. These findings illustrate the usefulness of multivariate approaches in revealing hidden spatial patterns and guiding more targeted public health interventions.

The article is structured as follows. The literature review section presents previous studies on the influence of climatic and structural factors on dengue transmission in Colombia. The materials and methods section details data sources, variables analyzed, and statistical methods employed. The results section presents the findings, including factor analysis, department grouping, and characterization of the identified clusters. The discussion section interprets these findings and examines their public health implications. Finally, the conclusions section summarizes the main points.

The literature has documented the influence of climatic and structural factors on dengue transmission in Colombia. One study analyzed the relationship between temperature, precipitation, and vegetation cover (normalized difference vegetation index, NDVI) in the department of Córdoba, between 2001 and 2010, finding that increased air temperature and decreased precipitation favor dengue occurrence. The results showed that the climatic variables also influence the occurrence of the disease, particularly when the socioeconomic conditions of the population are precarious ^7^.

From a broader perspective, Vanlerberghe and Verdonck addressed health inequality as a key determinant of dengue. They argued that impoverished populations bear a disproportionately high burden because they live in communities with limited infrastructure for water supply and waste disposal, conditions that facilitate vector reproduction. Likewise, limited access to protective measures such as repellents or physical barriers represents a structural disadvantage in prevention ^8^.

A study conducted in Palmira (Valle del Cauca) between 2010 and 2015 found significant correlations between dengue incidence and the climatic variables of temperature and precipitation. The authors emphasized that, although the results were conclusive regarding the role of climate variability, future research should incorporate cultural, demographic, and geographic variables for a more comprehensive understanding of the phenomenon ^9^. Along similar lines, another study modeled dengue transmission in Medellín using data from 2001 to 2011. The authors found a clear association between dengue transmission and local climatic conditions, particularly precipitation with a 20-week lag, showing how the effects of climate on transmission may manifest with time delays ^10^.

The study of Morgan *et al*. presented an integrated analysis of arbovirus cases reported between 2007 and 2017 and the local climatic and socioeconomic profiles of three Colombian municipalities (Bello, Cúcuta, and Moniquirá). Their results correlated climatic variables -such as average and minimum temperature, and wind speed- with disease transmission. Additionally, socioeconomic factors such as barriers to health and childhood care services, inadequate sanitation, and poor water supply indicated an unfavorable impact on dengue, Zika, and chikungunya transmission in the three ecosystems ^11^.

Finally, a systematic review synthesizing the findings of 97 articles, using the PRISMA (Preferred Reporting Items for Systematic Reviews and Meta-Analyses) approach, classified the determinants of dengue into environmental, social, and economic factors ^12^. A cross-sectional correlational study that showed a direct influence of physical and environmental household conditions on disease incidence is among the Colombian studies included ^13^. Another study, based on descriptive quantitative research, highlighted lack of education, inadequate management of water deposits and reservoirs, and limited access to public services as key social barriers ^14^. Overall, the systematic review concluded that socioeconomic factors -such as poverty, lack of education, poor hygiene habits, and scarcity of basic services (drinking water, sewage, and garbage collection)- significantly increase vulnerability to dengue ^12^.

## Materials and methods

### Studied departments

This study analyzed 31 administrative units (departments) in Colombia, including Chocó, Vaupés, Amazonas, Nariño, Guainía, Putumayo, Caquetá, Guaviare, Antioquia, Cauca, Caldas, Meta, Risaralda, Vichada, Santander, Valle del Cauca, Quindío, Córdoba, Casanare, Tolima, Norte de Santander, Bolívar, Arauca, Cundinamarca, Boyacá, Cesar, Huila, Sucre, Magdalena, Atlántico, and La Guajira. These departments vary substantially in terms of geographic conditions, infrastructure development, and access to public services.

According to Giles Álvarez *et al*., territories classified as vulnerable - mainly located in the Amazon, along with Chocó, and La Guajira- showed critical gaps in poverty, drinking water access, and sanitation ^15^. For example, these territories reported access to quality water sources up to 2.1 times lower than the national average and indoor sanitation conditions up to 1.9 times below the national average. These structural inequalities contrast with the so-called consolidated territories (Antioquia, Valle del Cauca, Quindío, Risaralda, Boyacá, Santander, Atlántico, and Cundinamarca), which have conditions above the national average across most analyzed indicators ^15^. Some of these departments exceed the national average in access to basic services by more than 30%, report monetary poverty levels 1.4 times lower than the national average, and show higher access to safe water sources and gross per capita production.

## Data collection on dengue incidence and socioeconomic conditions

We obtained the 2023 dengue incidence from the *Instituto Nacional de Salud* website ^16^, which provides detailed case reports at territorial level.

Regarding data related to basic services and socioeconomic conditions, the *Encuesta Nacional de Calidad de Vida* in Colombia, conducted by the *Departamento Administrativo Nacional de Estadística* (DANE) ^17^, is a fundamental tool for collecting socioeconomic data of households. The analysis of these conditions is key to investigating the relationship between access to public services and the incidence of diseases such as dengue, as previously reported. For example, in the study of Salim *et al*., in Guatemala, they observed that factors such as rapid population growth, mobility, poverty, and lack of basic services (access to safe water and sanitation) contributed to dengue transmission ^6^. Similarly, in the study of Zellweger *et al*., in New Caledonia, a higher dengue incidence was consistently associated with lower socioeconomic status, including higher unemployment rates and lower income ^18^.

Since the *Encuesta Nacional de Calidad de Vida* provides detailed information on access to services, such as drinking water and sanitation, we can correlate these data with the number of dengue cases in Colombia. Lack of access to running water, for example, forces people to store water in containers, creating ideal conditions for breeding *Ae. aegypti*, the main vector of dengue. By analyzing sections of the survey such as “households by access to public services”, it is possible to delve into how infrastructure conditions and population density influence dengue transmission.

### Climatic data

Based on previous studies ^19^^-^^21^, we defined the following climatic variables for 2023: maximum, minimum, and mean temperature; total accumulated precipitation; and the normalized difference vegetation index (NDVI). Data were extracted from open-access satellite sources. Images were delimited to the coordinate bounds of Colombia (longitude 81.84153° to 66.83774° W; latitude 4.228429° S to 15.91248° N).

Maximum, minimum, and mean temperature data were obtained from the Copernicus Climate Change Service (C3S) repository ^22^ under the ERA5 section. Temperature values, expressed in Kelvin degrees, were converted to Celsius degrees. Daily precipitation data were collected from CHIRPS (Climate Hazards Group InfraRed Precipitation with Station data) ^23^, with a spatial resolution of 0.05 km. Total cumulative precipitation was calculated and then averaged by department. NDVI information was obtained from the National Centers for Environmental Information ^24^, also at a 0.05 km resolution. Altitude data (digital elevation model) were sourced from the R geodata library ^25^. For NDVI and altitude, we calculated departmental averages using polygon-based spatial aggregation.

### Statistical methods

Due to the multidimensionality of the variables considered -climatic, socioeconomic, epidemiologic, and infrastructure-related- multiple factor analysis to reduce and identify the main latent structures within the dataset was done. This technique enables the integration of heterogeneous variable groups while preserving their internal correlations. Subsequently, hierarchical clustering methods and self-organizing maps were used to classify departments with structurally similar profiles. These methodologies were selected for their potential to reveal hidden patterns and to classify regions based on shared characteristics associated with dengue risk. Together, these tools favored a structured classification of departments, allowing the identification of territorial clusters with similar vulnerability characteristics.

- *Multiple factor analysis*. It was performed following the methodological guidelines described to detect underlying patterns and reduce dimensionality of the data ^26^. The adequacy of the variables for this analysis was assessed through the Kaiser-Meyer-Olkin test. In the analysis, the retained dimensions captured factors associated with climate, socioeconomic characteristics, and dengue prevalence.
- *Cluster analysis and self-organizing maps*. To delve into the differentiation between departments, we performed a hierarchical clustering analysis, using the FactoMineR package ^27^, based on the results of the multiple factor analysis. We complemented this analysis with self-organizing maps, originally introduced by Kohonen ^28^. As described by Carpio Martín ^29^, these maps are an unsupervised learning technique whose objective is to represent high-dimensional data sets in a coordinate system, so that points that are close in the original space remain close in the reduced-dimensional space. We configured self-organizing maps with a 3 by 1 hexagonal grid to visualize the relationships among departments and confirm cluster consistency. The hexagonal topology enhances unit distribution across the feature space, improving spatial representation. In addition, the selected grid maintains a balance between resolution and simplicity, allowing identification of territorial groupings without unnecessary complexity.

## Results

**Figure 1:**
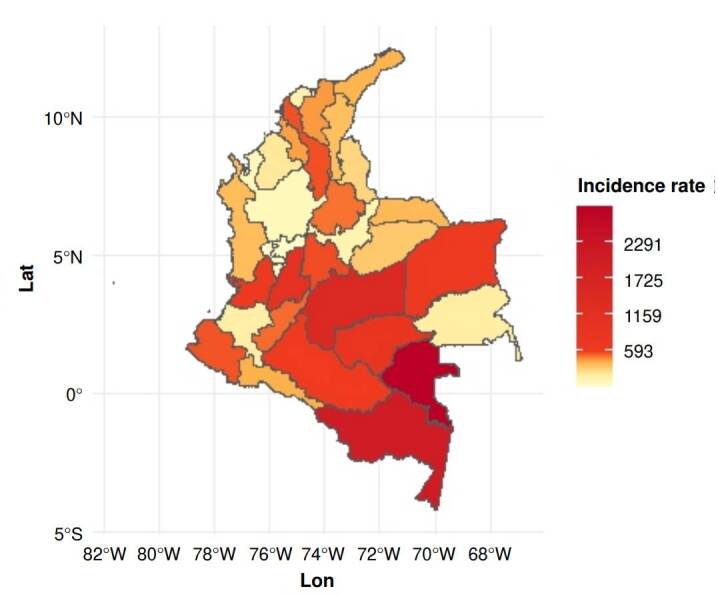
Dengue incidence rate per 100.000 inhabitan

Figure 1 shows a notable variation in dengue incidence across departments. The areas in yellow show low incidence levels, whereas those in red and brown indicate higher case burdens. The southeastern region of the country stands out as the most affected, in contrast to less impacted areas represented by lighter colors.

**Figure 2:**
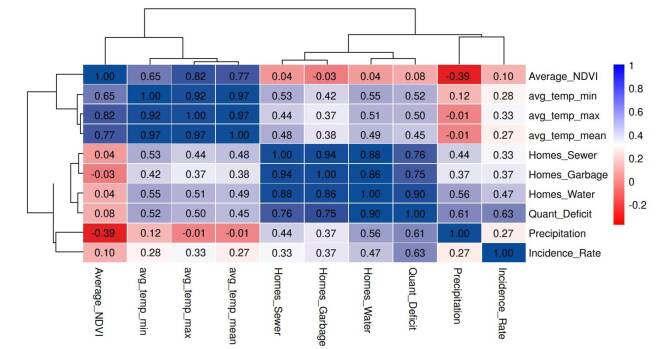
Correlation matrix

### Correlation matrix

Figure 2 shows the correlation matrix of the variables considered, revealing a strong positive association (> 0.70) between the indicators of access to basic services, homes water, homes sewer, and homes garbage. This pattern indicates that homes lacking one service generally do not have access to the others. The strong relationship between these variables and the quantitative housing deficit further suggests that limited access to basic services is an important indicator of structural deprivation in certain households.

Dengue incidence rate shows low positive correlations with access to water, sewage, and garbage collection (0.33 - 0.47), suggesting that the lack of these services may be associated with higher incidence. The strongest correlation was observed with the quantitative housing deficit (0.63), suggesting that inadequate housing conditions may favor transmission. Likewise, the weak correlation with precipitation (0.27) could indicate rainfalllimited influence on increased cases compared to housing-related factors.

With respect to the dendrogram shown in the correlation matrix, we found that one of the main clusters included the variables related to basic services: home water, home sewer, home garbage, and quantitative housing deficit. The proximity of these variables indicates that housing conditions are closely linked to access to or lack of basic services. In contrast, climate variables, such as average temperature minimum, maximum, and mean, form a separate group within the dendrogram. This pattern suggests that temperature-related conditions are interrelated independently of housing conditions.

The incidence rate occupies an intermediate position in the dendrogram, showing moderate correlation with climatic variables and housing conditions. This result indicates that, although dengue incidence may be influenced by temperature patterns and access to basic services, as previously reported ^6^, the strength of these associations is not enough to cluster closely with either group. Instead, its closest clustering is with basic services and quantitative housing deficit, indicating that inadequate housing conditions and limited access to basic services may highly influence dengue incidence.

### Multiple factor analysis

First, the Kaiser-Meyer-Olkin sampling adequacy test yielded an overall value of 0.78, indicating that the dataset was suitable for multiple factor analysis. The individual Kaiser-Meyer-Olkin values for the variables ranged from 0.70 to 0.85. Home water (0.85), home sewer (0.80), home garbage (0.80), and quantitative housing deficit (0.80) were the variables with the highest fit. High Kaiser-Meyer-Olkin values indicate potentially strong correlations with other variables and are useful for identifying underlying factors.

In contrast, variables such as precipitation and average NDVI had lower Kaiser-Meyer-Olkin values (around 0.70), indicating that although their inclusion in the analysis is acceptable, their contribution to factor identification may be limited. However, since all Kaiser-Meyer-Olkin values are above 0.70, the variable groups -climate, dengue, services, and deficit- were retained for factor analysis to characterize the interrelationships among these dimensions.

**Figure 3:**
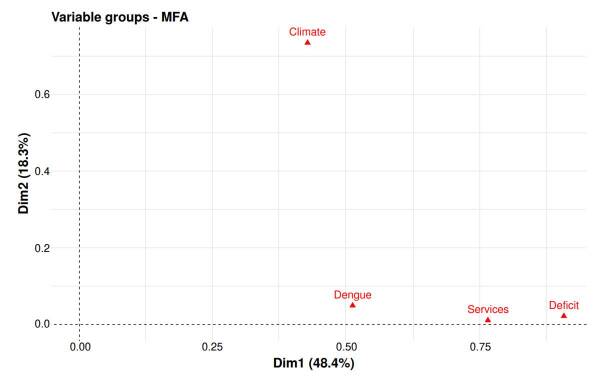
Factorial plan

Figure 3 displays the factorial plane for the groups of variables projected on the first two principal axes, which explain 76.7% of the total variability. The climate group is more distant along axis two, suggesting a pattern distinct from the other dimensions. Services, deficit, and dengue groups cluster more closely and are distributed toward axis one, indicating that dengue incidence is more associated with socioeconomic factors and housing conditions.

**Figure 4:**
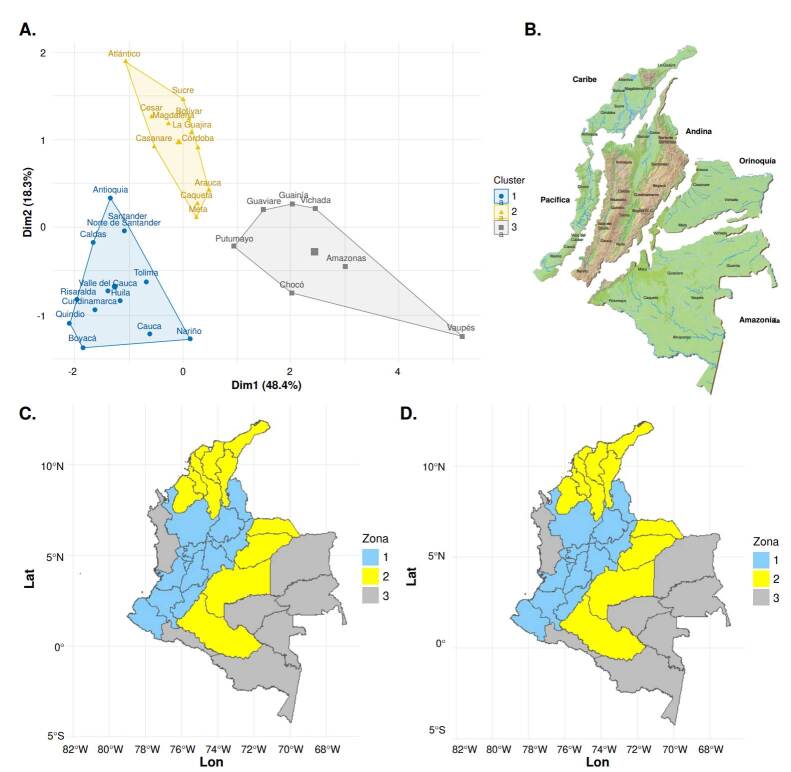
Comparison of analysis methods. **A)** hierarchical cluster; **B)** map of natural regions. Source: *Departamento Administrativo Nacional de Estadística* (2024); **C)** Multiple factor analysis cluster map; and **D)** self-organizing map

Figure 4a shows that departments such as Vaupés, Amazonas, and Vichada are located on the right side of axis one, which may indicate high levels of housing deficits and limited access to basic services. Conversely, departments such as Cundinamarca, Valle del Cauca, and Risaralda appear on the left side of the axis, associated with better infrastructure and access to services.

### Cluster analysis

Figure 4c  shows the grouping of departments based on the first five axes of the multiple factor analysis. Complementarily, a self-organizing map was constructed using the same variables (figure 4d) in a 3 by 1 hexagonal grid that allowed three main clusters to be identified. After normalizing the data, each department was assigned to a neuron representing similar multidimensional profiles. The self-organizing maps results were consistent with those obtained through hierarchical clustering, reinforcing the robustness of the groupings.

Group 1 (blue) comprises Tolima, Antioquia, Norte de Santander, Boyacá, Huila, Risaralda, and Cundinamarca departments, located mainly in the Andean region (figure 4b). This region is characterized by a bimodal climate with two rainy seasons ^30^ and great socioeconomic diversity. It accounts for 38.5% of the national population and 46.5% of Colombia’s gross domestic product (2020), and hosts nearly 448,000 companies ^31^.

Group 2 (yellow) includes the Caribbean departments -Atlántico, La Guajira, Sucre, Cesar, Magdalena, Bolívar, and Córdoba- (figure 4b), characterized by hot and humid climates, as well as arid zones and a diverse geography that supports agriculture, extensive livestock farming, and mining ^32^. This group also includes the Orinoquía departments: Arauca, Casanare, Meta, and Caquetá, whose warm and rainy climates, along with vast plains and forested areas ^32^, favor similar economic activities. These shared climatic, geographic, and productive characteristics explain their clustering into a common group.

Group 3 (gray) includes the departments of Guaviare, Guainía, Amazonas, Putumayo, and Vaupés, all located in the Amazon region and recognized for their high biodiversity and tropical rainforest ecosystems ^30^. This group also comprises Chocó, in the Pacific region, which has the highest annual rainfall in Colombia and a remarkable ecosystem complexity ^33^. The clustering of these departments is largely explained by their shared climatic and environmental characteristics.

Table 1 shows that variables such as temperature exhibit a strong relationship with the classification of the groups (η^2^ > 0.80), highlighting the predominant role of climatic conditions in differentiating departments. A significant association is also observed with access to basic services, especially to drinking water. In contrast, the dengue incidence rate shows a weaker association (η2 = 0.212), suggesting that, although relevant, it does not constitute the primary distinguishing factor among the groups.

**Table 1:** Relationship between variables and group composition

Variable	η2	p value
Average temperature means	0.843	5.164 x 10^-12^
Average temperature maximum	0.829	1.736 X 10^-11^
Home water	0.809	8.143 X 10^-11^
Quantitative housing deficit	0.806	1.034 X 10^-10^
Average temperature minimum	0.800	1.536 X 10^-10^
Home sewage	0.533	2.188 X 10^-5^
Average NDVI	0.512	4.273 X 10^-5^
Home garbage	0.456	1.948 X 10^-4^
Precipitation	0.456	1.973 X 10^-4^
Incidence rate	0.212	3.502 X 10^-2^

NDVI: Normalized difference vegetation index

According to table 2, group 1 (blue) has an average dengue incidence rate of 362.11 cases per 100,000 inhabitants, with high variability (SD = 300.15), and a range from 27.30 to 1,029.97 cases, indicating heterogeneous risk levels. Several factors may contribute to this variability, including disparities in access to basic services: On average, 20.34% of households lack garbage collection (maximum = 55%), 25.69% lack sewerage (maximum = 57.09%), and 8.98% lack access to drinking water (maximum = 25.52%). The average quantitative housing deficit is 5.78%, with a maximum of 20.32%.

**Table 2:** Descriptive statistics of group 1

Variable	Mean	Standard deviation	Minimum	Median	Maximum
Average NDVI	0.22	0.06	0.15	0.19	0.31
Average temperature maximum (°C)	27.45	2.26	23.21	26.97	30.78
Average temperature mean (°C)	18.97	2.13	15.30	18.80	22.22
Average temperature minimum (°C)	12.03	2.53	6.88	12.23	15.83
Home garbage	20.34	15.26	5.05	14.14	55.00
Home sewage	25.69	15.63	9.35	19.82	57.09
Home water	8.98	7.23	3.25	6.49	25.52
Incidence rate	362.11	300.15	27.30	296.93	1,029.97
Precipitation	2,318.12	598.22	1,575.13	2,185.08	3,766.86
Quantitative housing deficit	5.78	5.15	0.93	3.92	20.32

NDVI: Normalized difference vegetation index

Regarding climate, the average maximum temperature was 27.45 °C (SD = 2.26 °C) and the average minimum temperature was 12.03 °C (SD = 2.53 °C), with annual precipitation averaging 2,318.12 mm (SD = 598.22 mm). The average normalized difference vegetation index value was 0.22 (SD = 0.06), suggesting moderate vegetation cover.

Table 3 shows that group 2 (yellow) has an average dengue incidence rate of 493.44 cases per 100,000 inhabitants, with high variability (SD = 349.32) and a range from 159.94 to 1,475.88 cases, indicating concerning levels in several departments. This group presents greater deficiencies in basic services: on average, 26.69% of households lack garbage collection (maximum = 48.85%), 38.29% lack sewerage (maximum = 56.43%), and 18.51% have no access to drinking water (maximum = 45.36%). The average quantitative housing deficit is 12.64% (maximum = 30.08%).

**Table 3:** Descriptive statistics of group 2

Variable	Mean	Standard deviation	Minimum	Median	Maximum
Average NDVI	0.33	0.05	0.27	0.31	0.41
Average temperature maximum (°C)	34.77	0.74	33.57	34.78	36.03
Average temperature mean (°C)	26.30	0.96	24.89	26.37	27.89
Average temperature minimun (°C)	19.72	1.70	17.32	19.28	23.20
Home garbage	26.69	12.75	4.64	24.51	48.85
Home sewage	38.29	15.34	10.30	45.87	56.43
Home water	18.51	11.40	3.69	16.16	45.36
Incidence rate	493.44	349.32	159.94	406.03	1475.88
Precipitation	1,830.01	670.02	794.34	1,899.89	3,081.76
Quantitative housing deficit	12.64	8.11	3.08	9.86	30.08

NDVI: Normalized difference vegetation index

Climatically, these departments are characterized by warm conditions, with average maximum temperatures of 34.77 °C (SD = 0.74 °C), minimum temperatures of 19.72 °C (SD = 1.70 °C), and an average temperature of 26.30 °C. Average annual precipitation reaches 1,830.01 mm (SD = 670.02 mm), suggesting an intermediate rainfall regime with marked variability. The average normalized difference vegetation index value was 0.33, reflecting moderate vegetation cover.

Table 4 indicates that group 3 (gray) has the most limited access to basic services. On average, 53.68% of households lack garbage collection (maximum = 75.60%), 69.05% lack sewage (maximum = 98.19%), and 61.55% have no access to drinking water (maximum = 76.46%). The quantitative housing deficit is also the highest among all groups, averaging 51.20% and a maximum of 84.29%, indicating severely precarious housing conditions. The average dengue incidence rate in this group is 1,046.87 cases per 100,000 inhabitants, with high variability (SD = 991.08) and a range from 204.85 to 2,842.92 cases. Climatic conditions are warm and relatively stable, with an average maximum temperature of 34.61 °C, a minimum of 19.77 °C, and a mean of 25.51 °C. This group is also characterized by very high rainfall, averaging 3,423.52 mm (SD = 848.21 mm), consistent with predominantly humid environments.

**Table 4:** Descriptive statistics of group 3

Variable	Mean	Standard deviation	Minimum	Median	Maximum
Average NDVI	0,28	0,04	0,20	0,29	0,31
Average temperature maximum (°C)	34,61	1,59	31,93	35,24	36,21
Average temperature mean (°C)	25,51	0,97	23,95	25,63	26,85
Average temperature minimum (°C)	19,77	0,99	17,77	20,26	20,49
Home garbage	53,68	17,48	32,57	50,54	75,60
Home sewage	69,05	19,07	44,70	70,68	98,19
Home water	61,55	13,90	40,93	64,91	76,46
Incidence rate	1,046,87	991,08	204,85	661,32	2,842,92
Precipitation	3,423,52	848,21	2,399,30	3,321,30	5,112,19
Quantitative housing deficit	51,20	15,37	38,03	46,89	84,29

NDVI: Normalized difference vegetation index

As a representative example of group 1 (blue), the department of Risaralda has the lowest incidence rate among all the territories analyzed, with 27.3 cases per 100,000 inhabitants, placing it in a relatively low-risk category. This low incidence rate may be related to robust infrastructure and service coverage: 96.71% of households have access to drinking water, 85.9% to sewage, and 91.5% to garbage collection. The quantitative housing deficit was also low (3.26%), indicating adequate living conditions for most of the population. Climatically, Risaralda is in an intermediate zone with moderate temperatures (average maximum = 25.8 °C; minimum = 11.9 °C) and annual precipitation of 2,454 mm. This combination of temperate climate and extensive access to basic services may help limit the optimal conditions for the proliferation of *Aedes aegypti*. Its normalized difference vegetation index value (0.18) indicates moderate vegetation, possibly associated with a balance between urban and periurban areas. The case of Risaralda reinforces the idea that strengthened infrastructure and improved urban quality of life can serve as protective factors against dengue, even in regions with humid climates.

In group 2 (yellow), the department of Meta exemplifies territories with a high incidence of dengue, despite relatively favorable coverage of basic services. Meta reports the highest incidence rate within its group: 1,475.88 cases per 100,000 inhabitants, indicating a notably alarming epidemiological situation. Structurally, Meta displays adequate service coverage since 98.4% of households have electricity, 83.8% have access to drinking water, 77.8% to sewage, and 85.9% to garbage collection. The quantitative housing deficit stands at 8.32%. However, these conditions have not prevented significant dengue transmission. Meta has a warm humid climate typical of endemic areas, with an average temperature of 25.2 °C, maximum temperatures approaching 33.92 °C, and accumulated annual precipitation of 2,683 mm, conditions highly favorable for *Ae. aegypti* proliferation. The normalized difference vegetation index value (0.28) indicates moderate vegetation cover, which may contribute to vector persistence. The case of Meta illustrates how a high incidence of dengue can be driven by climatic and ecological factors, even in the presence of adequate basic infrastructure.

In group 3 (gray), Chocó represents a case of marked structural vulnerability. This department is characterized by an extremely humid climate, with an average annual rainfall exceeding 5,100 mm, one of the highest in Colombia. Temperatures remain warm throughout the year, creating highly favorable conditions for the proliferation of *Ae. aegypti*. Structurally, Chocó faces severe deficiencies in access to basic services: only 28.4% of households have access to drinking water, 16.2% to sewerage systems, and 49.5% to garbage collection. The quantitative housing deficit reaches an alarming 46.9%. Although electricity coverage is relatively high (95.5%), overall housing conditions are precarious. The dengue incidence rate of 377.12 cases per 100,000 inhabitants reflects how the combination of extreme environmental factors (high humidity and persistent warmth) and structural deprivation considerably increases transmission risk.

Taken together, these three cases highlight how different combinations of structural and environmental factors shape distinct dengue epidemiological profiles. Risaralda exemplifies a territory where robust infrastructure and temperate climate may help reduce vulnerability. Meta demonstrates that, even with relatively adequate service provision, ecological suitability can drive high transmission. Chocó, in turn, exemplifies how severe structural vulnerability in an environmentally favorable setting for the vector can amplify risk, particularly where deficiencies in water and sanitation act as direct facilitators of *Ae, aegypti* proliferation and dengue transmission.

## Discussion

This study shows that dengue incidence in Colombia may be driven by the interaction between structural, climatic, and ecological factors. Using multiple factor analysis, correlation patterns, and departmental groupings derived from hierarchical clustering and self-organizing maps, three territorial and contrasting profiles were identified to help understand the distribution and determinants of risk. While Risaralda exemplifies a territory with robust infrastructure and a low risk of transmission, Meta exhibits a high incidence rate despite relatively favorable service coverage, reflecting a marked influence of ecological and climatic conditions. In turn, Chocó presents high structural vulnerability combined with extremely humid climatic conditions, making it a particularly exposed and high-risk territory.

These findings have key implications for the formulation of public policies aimed at controlling dengue. Strategies must go beyond an exclusively health-based approach and incorporate the structural determinants of the habitat, promoting sustained improvements in basic infrastructure, access to essential services, and urban planning with a preventive focus. In addition, a differentiated territorial approach is required to address the predominant factors in each context: in departments with high structural vulnerability, investments should prioritize access to drinking water and sanitation; in territories with ecological conditions favorable to the vector, environmental control measures need to be reinforced; and in regions with more consolidated infrastructure, good urban practices and risk-management strategies should be promoted.

The results of this study are broadly consistent with previous scientific literature, which has demonstrated the influence of structural and climatic factors on dengue dynamics. For example, Kenneson et al. found in Ecuador that access to safe drinking water, use of mosquito nets, and indoor fumigation reduced the risk of infection ^34^. Similarly, Schmidt et al., in Vietnam, identified lack of access to safe drinking water as a key predictor of dengue risk, especially in areas with population densities of 3,000-7,000 inhabitants/km^2 (^^35^; they also reported higher dengue risk in rural than in urban areas. Gibb et al. showed that local factors, like infrastructure conditions and urban sprawl, influence dengue risk more than variables such as temperature or mobility ^36^. They found that dengue risk increased with slightly improved access to hygienic toilets but tended to decrease as more households had access to safe drinking water.

Thus, when only a portion of the population has access to these services, the risk may be higher, while broader coverage helps to reduce it. Furthermore, sustained urban growth over time was associated with a decreased risk, suggesting that cities with more structured planning and development may be better prepared to prevent dengue transmission. In contrast, factors such as traffic and mobility showed a positive association with dengue risk, but with lower predictive value compared to infrastructure. These findings indicate that not only climate and urban density explain the spread of dengue, but also the basic environmental conditions where populations live.

In the Colombian context, Overgaard et al. showed that water storage associated with supply deficiencies is a key factor in the proliferation of dengue and diarrheal diseases, due to the generation of breeding sites and the risk of contamination ^37^. Ortiz et al. warned that the combination of environmental degradation and poor infrastructure intensifies the risk of vector-borne diseases ^38^, and ACAPS confirmed this by documenting that, in Putumayo, climatic conditions, deforestation, and land use favor vectors’ habitats ^39^.

These findings align with Latin American studies in Costa Rica (5) and Guatemala (6), which highlight the role of poverty and limited access to safe water in the spread of dengue. In Colombia, Perdomo-Balaguera et al. found that illiteracy, lack of access to safe water, and overcrowding are significantly associated with dengue incidence ^40^. Although our study used different variables, housing shortages and access to services emerged as critical elements in the formation of the departmental clusters.

Finally, although the regression model reported a weak relationship between temperature and dengue ^40^, our findings show that variables such as minimum, mean, and maximum temperatures were decisive in department clustering, especially in group 3, which presents the highest temperatures and the highest incidence rates, consistent with Rey-Ardmirola et al. proposal ^41^.

As a future line of research, we recommend studying and including more disaggregated indicators at a municipal or smaller unit level, as well as integrating real-time climate data to strengthen predictive modeling and early warning systems. Furthermore, incorporating variables such as seasonal vegetation cover, human mobility, and microenvironmental conditions could allow for better risk characterization.

### Strengths

This study applied analysis tools such as multiple factor analysis and self-organizing maps, which enabled robust dimensionality reduction and classification of departments.

### Limitations

A possible bias is the use of multiple sources of information. In addition, the study is limited to 2023, which restricts the generalizability of the results to other periods.

### Implications for public health

The classification of departments by groups facilitates the identification of the most vulnerable territories, enabling the design of more targeted policies. These findings are relevant for guiding dengue prevention and control strategies, optimizing available resources.
